# In silico prediction suggests inhibitory effect of halogenated boroxine on human catalase and carbonic anhydrase

**DOI:** 10.1186/s43141-022-00437-x

**Published:** 2022-11-03

**Authors:** Tarik Corbo, Abdurahim Kalajdzic, Dzelila Delic, Sumaia Suleiman, Naris Pojskic

**Affiliations:** 1 Institute for Genetic Engineering and Biotechnology, University of Sarajevo, Zmaja od Bosne 8, 71000 Sarajevo, Bosnia and Herzegovina; 2 Faculty of Science, University of Sarajevo, Zmaja od Bosne 33 – 35, 71000 Sarajevo, Bosnia and Herzegovina

**Keywords:** Dipotassium-trioxohydroxytetrafluorotriborate, Halogenated boroxine, Human catalase, Human carbonic anhydrase, Inhibitory, In silico

## Abstract

**Background:**

This research work included bioinformatics modeling of the dipotassium-trioxohydroxytetrafluorotriborate-halogenated boroxine molecule, as well as simulation and prediction of structural interactions between the halogenated boroxine molecule, human carbonic anhydrase, and human catalase structures. Using computational methods, we tried to confirm the inhibitory effect of halogenated boroxine on the active sites of these previously mentioned enzymes. The three-dimensional crystal structures of human catalase (PDB ID: 1DGB) and human carbonic anhydrase (PDB ID: 6FE2) were retrieved from RCSB Protein Data Bank and the protein preparation was performed using AutoDock Tools. ACD/ChemSketch and ChemDoodle were used for creating the three-dimensional structure of halogenated boroxine. Molecular docking was performed using AutoDock Vina, while the results were visualized using PyMOL.

**Results:**

Results obtained in this research are showing evidence that there are interactions between the halogenated boroxine molecule and both previously mentioned proteins (human carbonic anhydrase and human catalase) in their active sites, which led us to the conclusion that the inhibitory function of halogenated boroxine has been confirmed.

**Conclusion:**

These findings could be an important step in determining the exact mechanisms of inhibitory activity and will hopefully serve in further research purposes of complex pharmacogenomics studies.

## Background

Protein interactions have an important role in detecting the function of proteins and pathways in biological processes. It is of great interest and importance to determine protein-protein interaction in order to understand the process at the molecular level [1]. Prediction of the interaction between small molecules and proteins is a crucial step that sheds light on many biological processes, and plays an important role in the discovery of new drugs [2]. Knowing the location of the protein binding site significantly increases the efficiency of docking analysis [3]. Docking can be performed through two interrelated steps: first by determining different ligand conformations in the active site of the protein, then by ranking those conformations using scoring functions [4, 5].

Boroxines are products of dehydration of organoboronic acids. Due to their unique electronic structure, these substances are currently being investigated as possible enzyme inhibitors and therapeutics. This field of intensive research culminated in the commercialization of peptidylboronic acid as the antineoplastic drug Velcade. K_2_(B_3_O_3_F_4_OH) is a boron inorganic derivative formed in the reaction of potassium hydrofluoride (KHF_2_) with boric acid, in a molar ratio of 2:3 [6].

Due to the lack of bioactivity research on halogenated boroxine (HB), a group of authors [7–13] conducted a study that tested the antiproliferative, cytotoxic, and genotoxic potential of dipotassium-trioxohydroxytetrafluorotriborate K_2_(B_3_O_3_F_4_OH) in human cell cultures. The results obtained by these analyses showed significant inhibitory effects of the tested concentrations of HB on cell growth in carcinoma cell cultures, which confirmed the antitumor potential [7, 10, 14–16].

Catalase (CAT) is known to be one of the important enzymes in the function of the organism and its protection from toxic substances, such as hydrogen peroxide [H_2_O_2_]. Catalase is an antioxidant enzyme that accelerates the decomposition reaction of hydrogen peroxide and thus protects the cell membrane and DNA from this harmful oxidation process. This reaction is important for the organism, because hydrogen peroxide is produced as a by-product of many normal cellular reactions, and the loss of catalase function is associated with increased sensitivity to oxidative stress [17]. Reactive oxygen species (ROS) have been shown to be toxic, but they can also react as signaling molecules. The resistance of tumor cells to intercellular ROS signals depends on the expression of catalase on the cell membrane. Intercellular ROS signals can be restored if hydrogen peroxide is provided and catalase is inhibited [18].

These findings defined the biochemical basis for the specific induction of apoptosis in tumor cells. This model represents a potential new approach in tumor prevention and treatment research. A study conducted by a group of authors [19] showed that HB inhibits the enzyme catalase, and thus leads to higher production of hydrogen peroxide and an increase in ROS signals. Applying a cream containing HB to the tumor site or injecting it inside the tumor would significantly reduce catalase activity and increase the number of hydrogen peroxide, and in that way, the tumor cells would take themselves into apoptosis. Further in vitro and in vivo studies are needed to confirm this hypothesis [19].

Carbonic anhydrases (CA) are metalloenzymes, which catalyze the hydration of carbon dioxide into bicarbonates and protons. These enzymes can be found in different organisms within the entire phylogenetic tree, as five different, genetically separated families, α, β, γ, δ, and ζ-CA. The metal ion from the active site of the enzyme (which could be Zn, Fe, Cd, or Co) is essential for catalytic activity and also, for the binding of most (but not all) classes of CA inhibitors, which have been investigated so far, such as inorganic anions, sulfonamides, and dithiocarbamates [20, 21]. Several CA isoforms, such as CA II, IX, and XII, are associated with tumors or are involved in tumorigenesis and metastasis. Carbonic anhydrases of sulfonamides, sulfamates, or coumarin type are effective alone or in a combination with anticancer agents, in reducing the growth of the primary tumor and its metastasis [22].

In a study conducted by a group of authors [15], the boron heterocyclic substance dipotassium-trioxohydroxytetrafluorotriborate was investigated as an inhibitor of the zinc ion of CA. Eleven human CA isoforms (CA I–IV, VA, VI, VII, IX, and XII–XIV) were included in the study. K_2_(B_3_O_3_F_4_OH) did not inhibit hCA III, an isoform characterized by low CO2 hydrase activity and the presence of numerous Phe residues. On the other side, isoforms of hCA VA, hCA VI, hCA IX, and hCA XIII were inhibited by halogenated boroxine in the submillimolar range. Also, isoforms of hCA I and II, hCA IV, hCA XII, and hCA XIV, in comparison with the previous group, were much more effectively inhibited by halogenated boroxin, and the hCA VII isoform was very effectively inhibited by K_2_(B_3_O_3_F_4_OH) [22].

This group of authors considered that K_2_(B_3_O_3_F_4_OH) binds to a metal ion from the active site of the enzyme. The authors also hypothesized in this article that one of the beneficial antitumor effects of halogenated boroxine may also be the inhibition of carbonic anhydrases present in tumors [22].

## Methods

The three-dimensional structures of human catalase protein and human carbonic anhydrase (hCA) were downloaded from RCSB PDB database (Research Collaboratory for Structural Bioinformatics Protein Data Bank), which was established as the first online database with free access.

This study required a 3D structure of catalase that was not reacting with other molecules, because it was important to obtain proper potential interactions with halogenated boroxine. Human catalase with accession number 1DGB was chosen for this study.

Also, a search was performed for available structures of human carbonic anhydrase in the RCSB PDB database. Most of the structures were preserved in interaction with other proteins or molecules, and for easier research, we chose an independent structure of human carbonic anhydrase IX with accession number 6FE2.

**Fig. 1:**
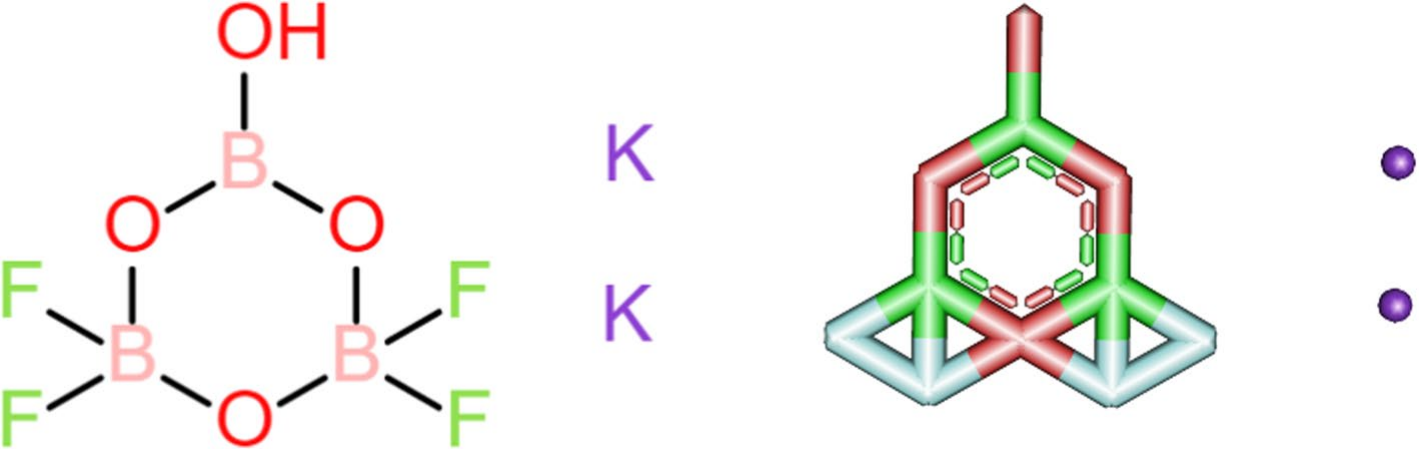
Visualization of two- and three-dimensional structure of halogenated boroxine

Two programs were used for creating a three-dimensional structure of halogenated boroxine: ACD/ChemSketch and ChemDoodle [23, 24]. Finally, the given molecule of halogenated boroxine is shown in Fig. 1.

After the molecule was drawn in a 2D sketcher, it was saved and visualized in ChemDoodle 3D, in different formats (pdb, sdf, xyz, mol, smiles, mol2), so that it can later be used in different software.

To predict the interaction between CAT, hCA, and HB, we used the AutoDock Vina software. Before that, we used AutoDock Tools to prepare the HB molecule, as well as the CAT and hCA protein, and save them in a special pdbqt format, which is recognized by AutoDock Vina. We first prepared the HB molecule, for which it was necessary to determine the rotating bonds and to preserve it in the previous mentioned format. The proteins also required certain changes, so we had to delete the water molecules, add polar hydrogen atoms, and assign Kollman charges. We saved the modified protein in the same folder as the molecule, which is important for the further use of the AutoDock Vina software [25]. Once both, the ligand and receptor were ready, docking analysis was initiated using the command line.

For the purpose of determining the exact site of interaction between the HB molecule and the two proteins, two different coordination fields were used, one for CAT and another one for hCA, presented in the form of a gridbox. In AutoDock Tools, the position and size of the gridbox are visually determined, and then, the coordinates were entered in a configuration file which AutoDock Vina can read.

AutoDock Vina is a program that does the docking in a space defined by coordinates, but within it, the results obtained cannot be visually inspected. Vina presents a table with the best possible results that the software has found and gives the coordinates for each result, which can be visually inspected in one of the visualization programs.

In terms of visualizing the results, we used PyMOL software [26]. After the docking analysis, the results were saved as a pdbqt file and they were inserted into the PyMOL software, together with the protein pdbqt file, in order to visualize the results. Each of the two files contained 9 results, which were analyzed one by one. For each of the results, a ligand site assay was performed, where we were able to see if the HB molecule interacted with the CAT and hCA at the given position.

## Results and discussion

**Fig. 2:**
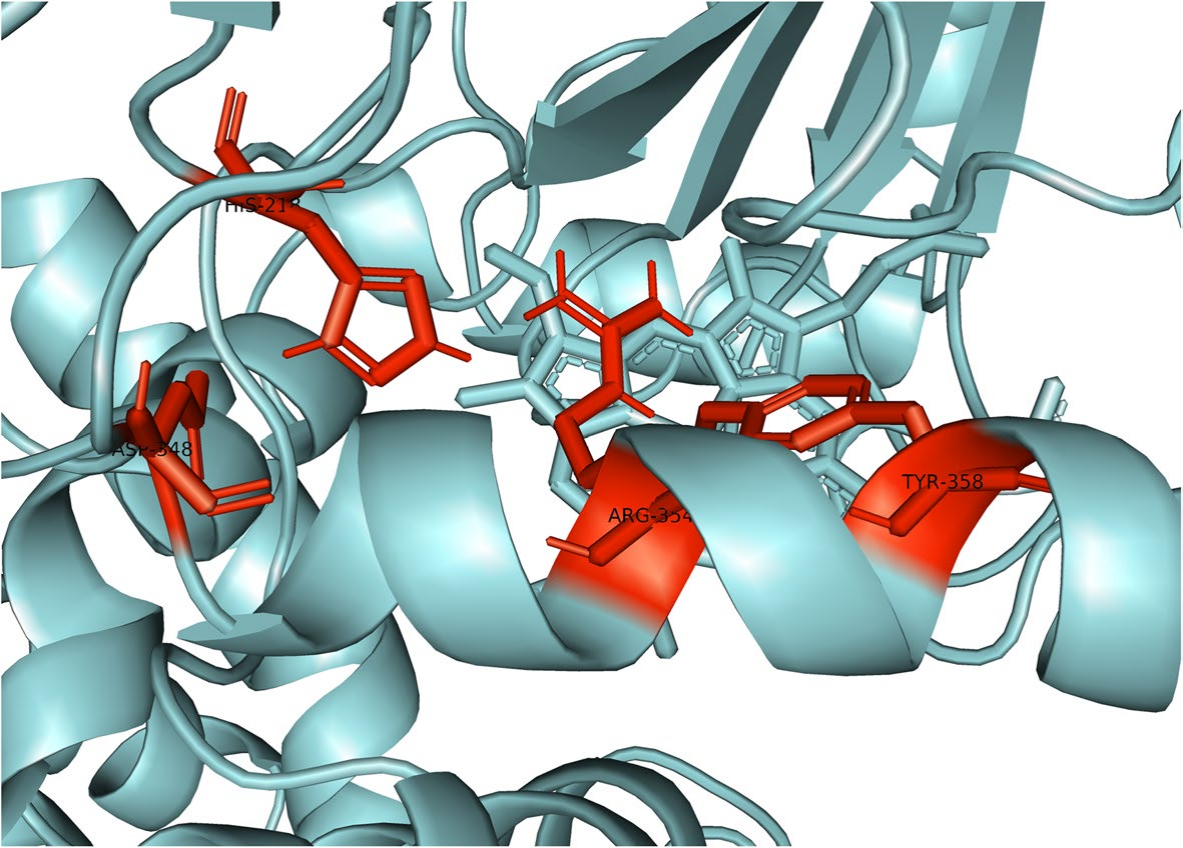
Active heme center of the catalase

We used two different gridboxes, one for the human catalase and one for the human carbonic anhydrase, and later, we compared the results. The gridbox dimensions regarding the CAT enzyme were 62 × 78 × 72 Å (ångstrom) and it was centered at 15, 20, 3. Value of spacing (ångstrom) was set to 1.0, and together with the *x*, *y*, and *z* coordinates determines the size of the gridbox. Using this gridbox, we sought to capture the active heme center of the enzyme catalase. The amino acids Tyr358, Arg354, His218, and Asp348 represent preserved residues for reaction with the ligand and may be analogs for Fe-His-Asp “triad” which is present in most peroxidases and Fe-Cys-Fe_4_S_4_ in sulfide reductases [27–29]. The labeled amino acids can be seen in Fig. 2. Morris et al. [30] considered that the gridbox system dependence is the main limit of AutoDock Vina program. A system without a gridbox would be effective in some cases, because it gives a dose of flexibility, but then two new problems would arise: calculating energy consumption would be much more sensitive and due to the greater conformational space, there would be a greater possibility of false-positive results [30].

**Fig. 3:**
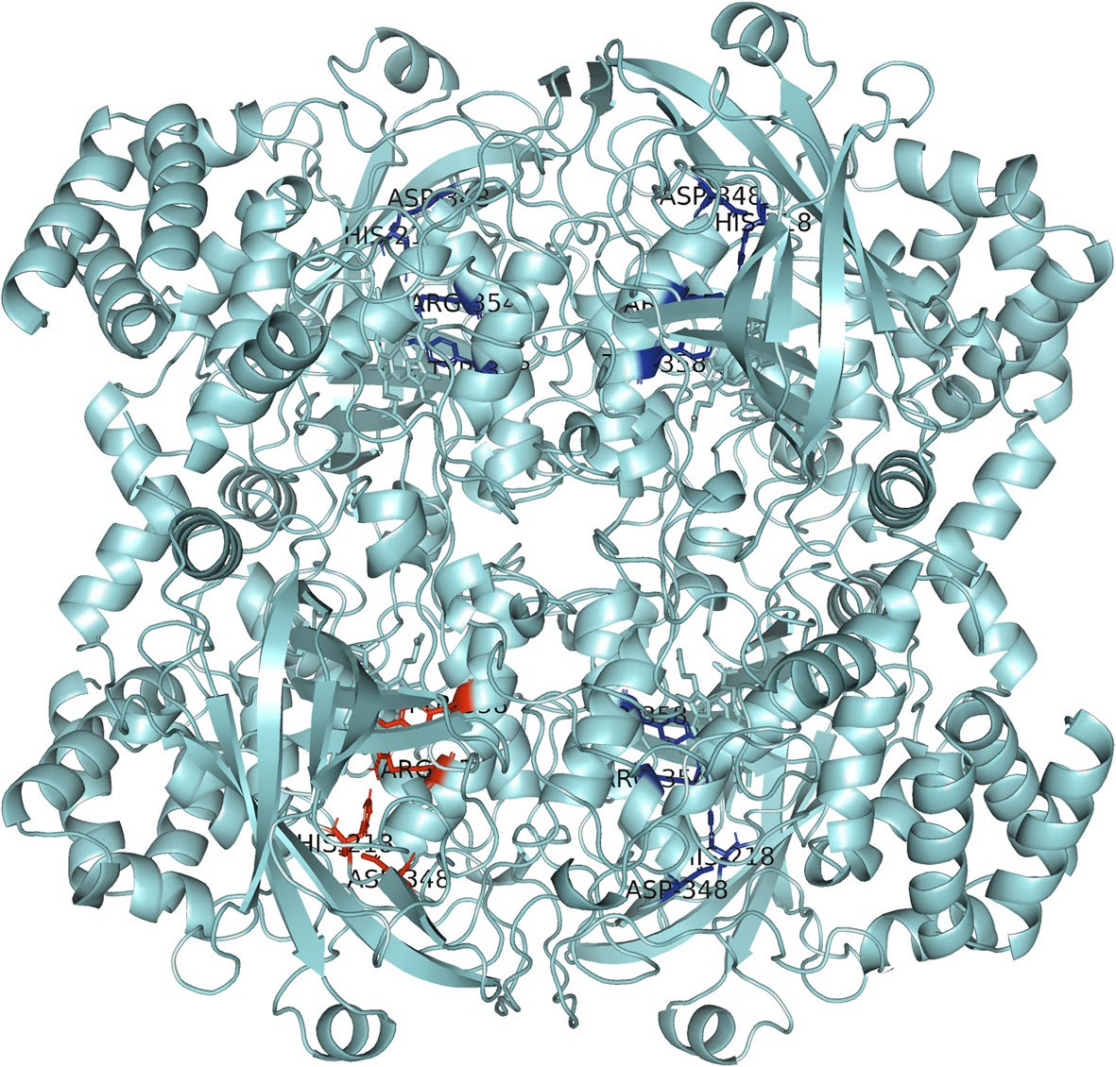
Active heme centers in all catalase subunits

Each of the aforementioned amino acids of the active site is present in all four catalase subunits, so that the gridbox encompasses all four active sites. The other three active sites can be seen in Fig. 3 and are highlighted in dark blue.

The results obtained with the docking analysis within the gridbox for CAT-HB interaction are shown in Table 1.

**Table 1:** Results of docking analysis in the AutoDock Vina software within the gridbox for CAT enzyme

Mode^a^	Affinity^b^ (kcal/mol)	Distance from best mode^c^	
		Rmsd l.b.	Rmsd u.b.
1	−6.2	0.000	0.000
2	−6.0	4.029	5.068
3	−6.0	33.091	34.244
4	−5.9	5.160	6.090
5	−5.9	1.937	2.919
6	−5.9	3.668	4.420
7	−5.8	35.074	36.231
8	−5.8	3.046	3.633
9	−5.8	2.860	3.907

^a^The result under ordinal number 1 is considered as the most relevant and the other results are presented in relation to it. ^b^The free energy expended in this interaction. ^c^The distance of other results from the best result

The result under ordinal number 1 is considered to be the most relevant and best one, and all other results are presented in relation to it. AutoDock Vina calculates the best result based on energy consumption and then compares each subsequent one with it; however, if the following results do not differ much from the first one, they should also be considered as potentially accurate [31]. The ordinal numbers of the obtained results are arranged in the “Mode” column and their order is based on the “Affinity” column, which shows the free energy released during the process of ligand-receptor interaction. The binding mode with the least binding energy is the most stable for the ligand and it actually represents the best mode of binding. The “Distance from best mode” column shows the distance of other results from the first, best result. RMSD (root-mean-standard-deviation) is the most commonly used unit of measurement for the quantitative determination of similarities between atomic coordinates and is expressed in ångström (Å) [32]. However, there are two different values of RMSD, RMSD l.b. (lower bound) and RMSD u.b. (upper bound), and these two differ in how atoms are denoted in distance calculations. RMSD u.b. examines each atom in one conformation with the same atom in another conformation, ignoring any symmetry. RMSD l.b. examines each atom in a particular conformation with the nearest atom of the same element in a different conformation [25]. As for the results, the first (−6.2 rmsd l.b. 0.000; rmsd u.b. 0.000) of them is, as we have already mentioned, the most relevant, and those that are closer to it in terms of the overall score are results under ordinal numbers: 2 (−6.0 rmsd l.b. 4.029; rmsd u.b. 5.068), 4 (−5.9 rmsd l.b. 5.160; rmsd u.b. 6.090), 5 (−5.9 rmsd l.b. 1.937; rmsd u.b. 2.919), 6 (−5.9 rmsd l.b. 3.668; rmsd u.b. 4.420), 8 (−5.8 rmsd l.b. 3.046; rmsd u.b. 3.633), and 9 (−5.8 rmsd l.b. 2.860; rmsd u.b. 3.907). The other two results, 3 (−6.0 rmsd l.b. 33.091; rmsd u.b. 34.244) and 7 (−5.8 rmsd l.b. 35.074; rmsd u.b. 36.231), are far more distant from the first, best conformation, so they are considered irrelevant. Also, conformations under the stated ordinal numbers, according to the rules, should not be taken into consideration, because they exceed the value of 2 Å. Only values below 2 Å are considered relevant and accurate [33, 34].

**Fig. 4:**
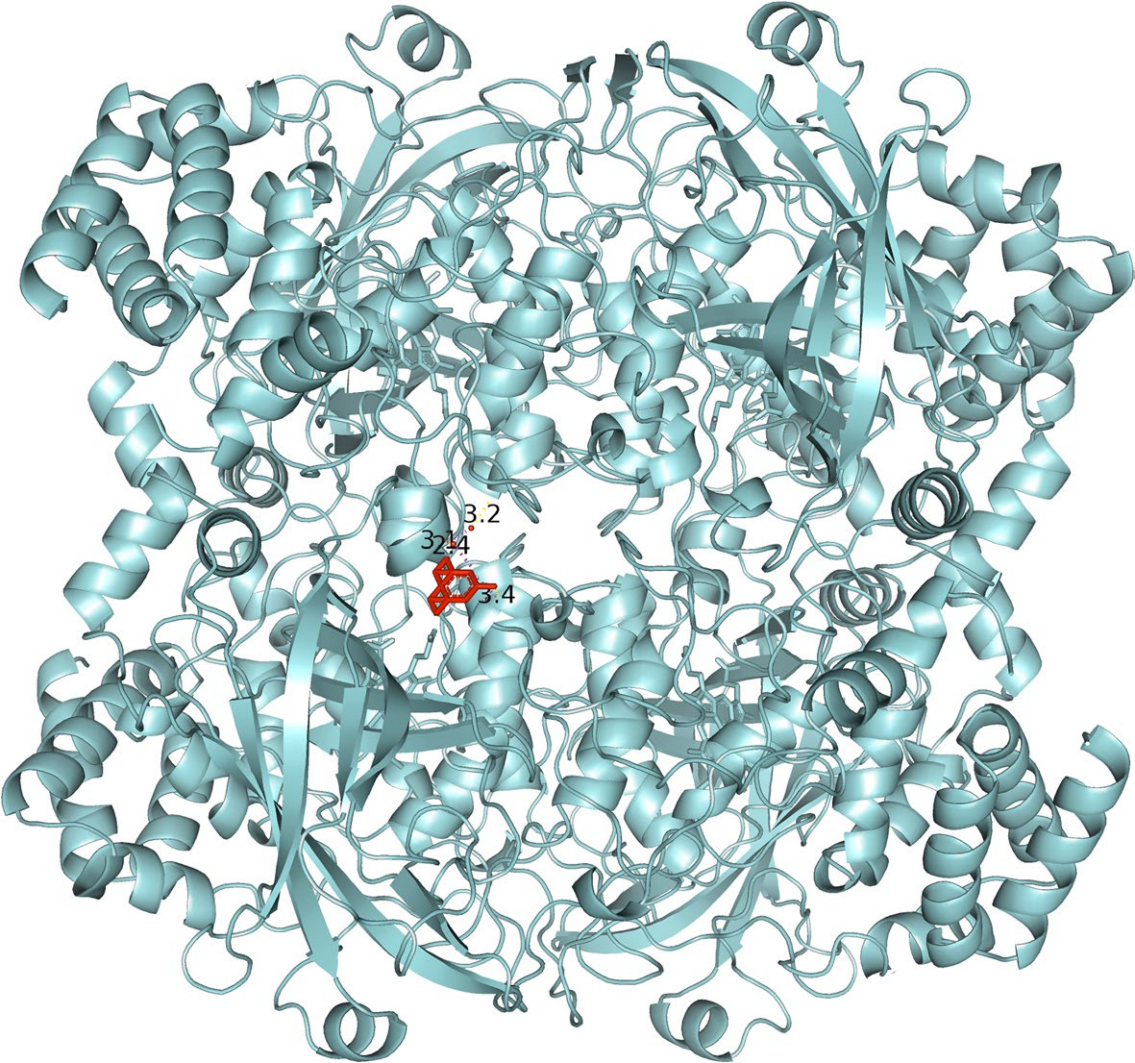
General overview of the interaction between halogenated boroxine molecule and humane catalase

The results obtained from the prediction of the interaction between HB and CAT were visually analyzed in PyMOL. The visual display gives us a better insight into the interaction and position of the molecule in regard to the protein. The first and only relevant result of the CAT gridbox is presented in Fig. 4.

**Fig. 5:**
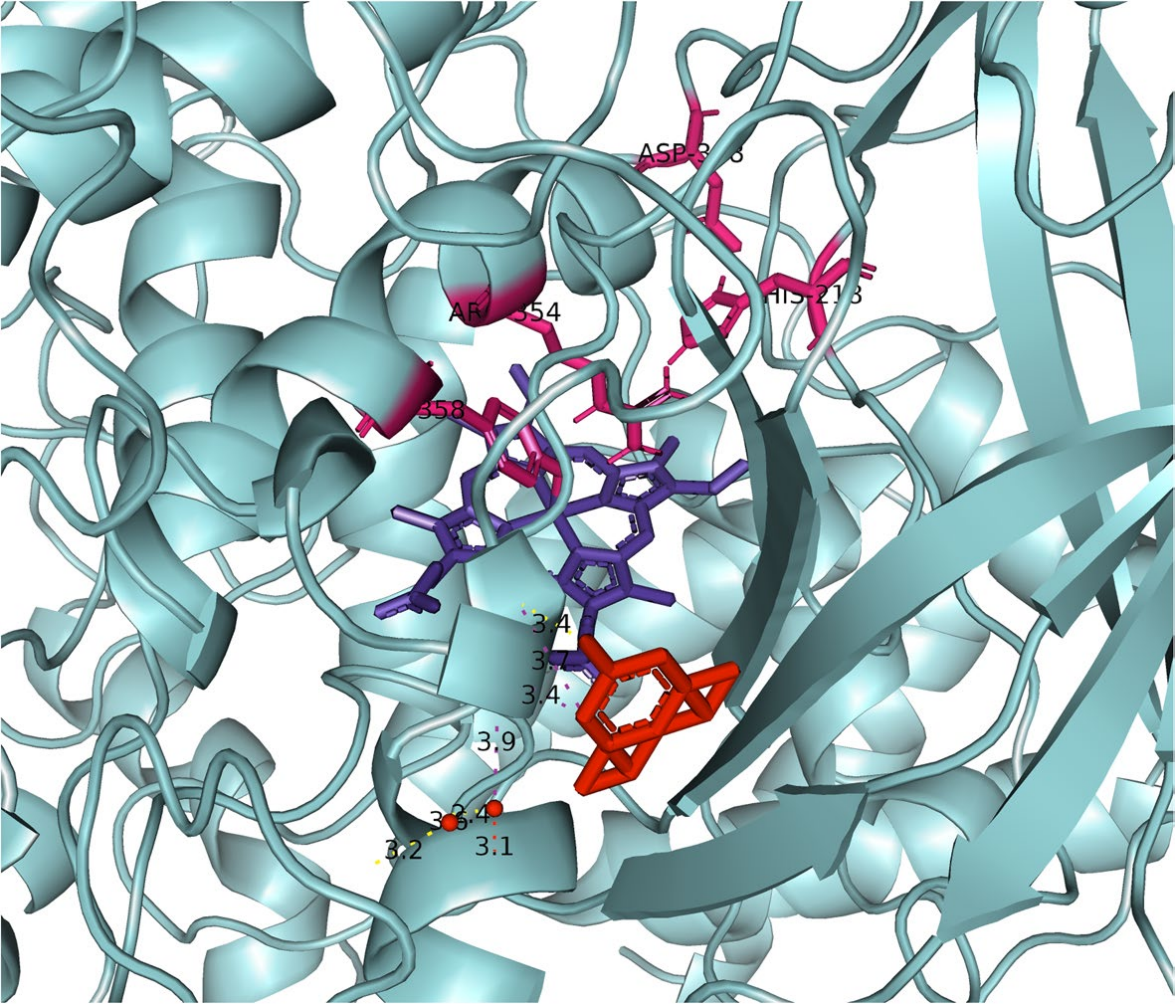
Closer look at the interaction between halogenated boroxine and human catalase

Figure 4 shows the interaction of HB and CAT. The HB molecule is marked in red, and the CAT molecule is marked blue, while the yellow-marked structures represent interaction sites. This figure gives us an overall insight into the binding site of the molecule to the protein and it shows that the molecule is near the active center of one domain of human catalase. Figure 5 will bring us closer to the place of interaction, and we will be able to explain it in more detail.

**Fig. 6:**
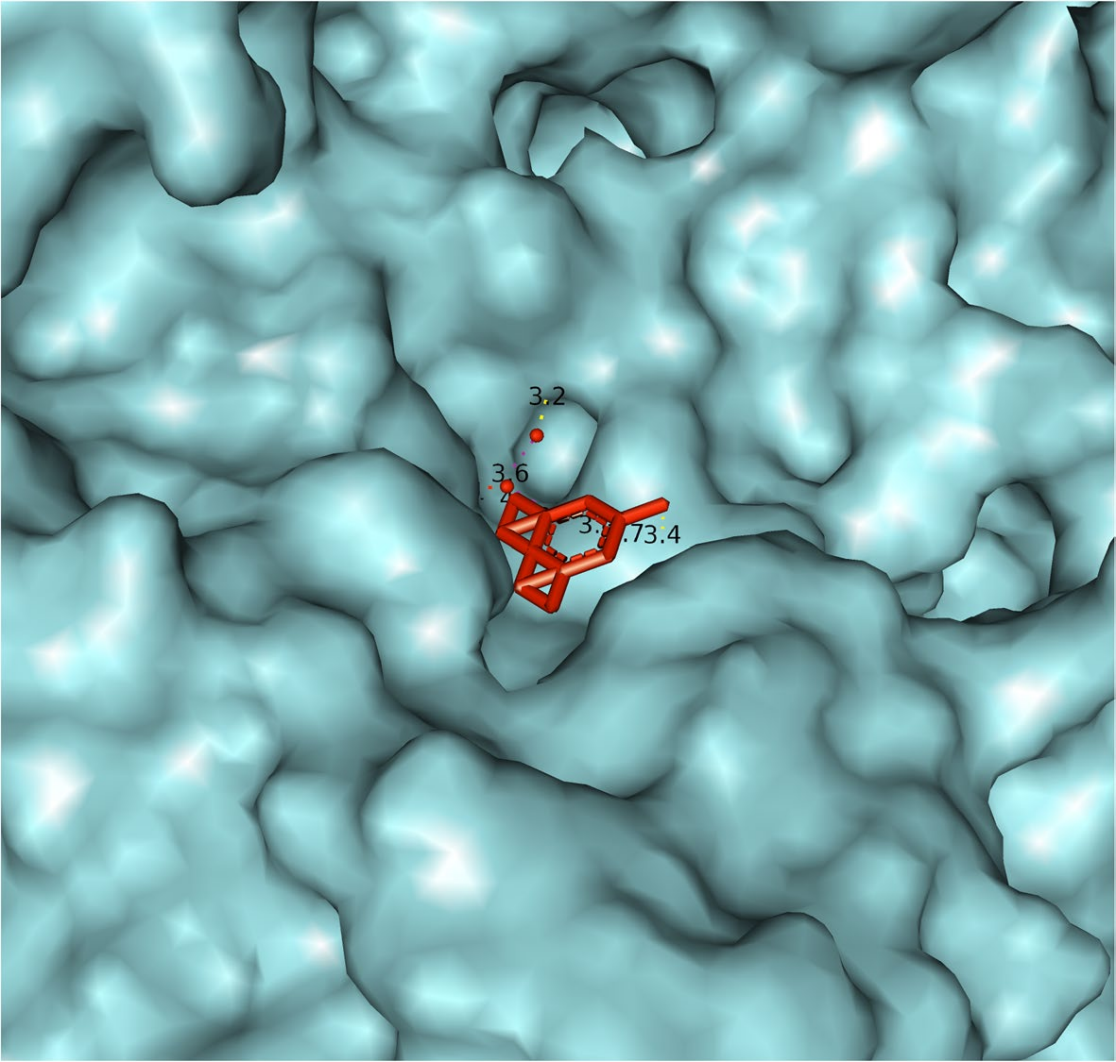
Pocket which contains the molecule of halogenated boroxine interacting with the heme group of human catalase

Figure 5 presents a closer view of the interaction between the HB molecule and the CAT protein. The HB molecule is marked in red, and the connections between atoms, i.e., the interaction between the molecule and the protein are marked in yellow, while the heme prosthetic group in the active center of the enzyme is marked in purple. The amino acids Tyr358, Arg354, His218, and Asp348 are marked in pink, which are preserved residues for ligand interaction, and which may represent analogues for the Fe-His-Asp “triad” which is present in most peroxidases and Fe-Cys-Fe_4_S_4_ in sulfide reductase [27–29]. Examining the ligand sites in PyMOL, these yellow dashed lines were obtained, which connect the sites of interaction between the atoms of the examined elements. Denoting the given atoms, it was discovered that the molecule of halogenated boroxine reacts directly with the heme group in the active center of the enzyme. This confirms our assumption that halogenated boroxine reacts with the human catalase protein, in its active site, and thus blocks the binding of other elements to catalase. Figure 6 shows the pocket containing the halogenated boroxine molecule interacting with the heme group.

In the previous figure, a pocket or funnel which contains the molecule of halogenated boroxine interacting with human catalase can be seen. The heme group is hidden under the sheet, but yellow dashed lines can be seen leading to it. With this research, we confirmed that halogenated boroxine reacts with human catalase in its active center, i.e., that it has an interaction with the heme group. The other results that we obtained, which are irrelevant, as we mentioned earlier, were listed only for comparison.

On the other side, the gridbox we used for hCA had dimensions 22, 24, and 28, centered at −24.6, −9.2, and 3.1. The value of spacing (ångstrom) was set to 1.0.

**Fig. 7:**
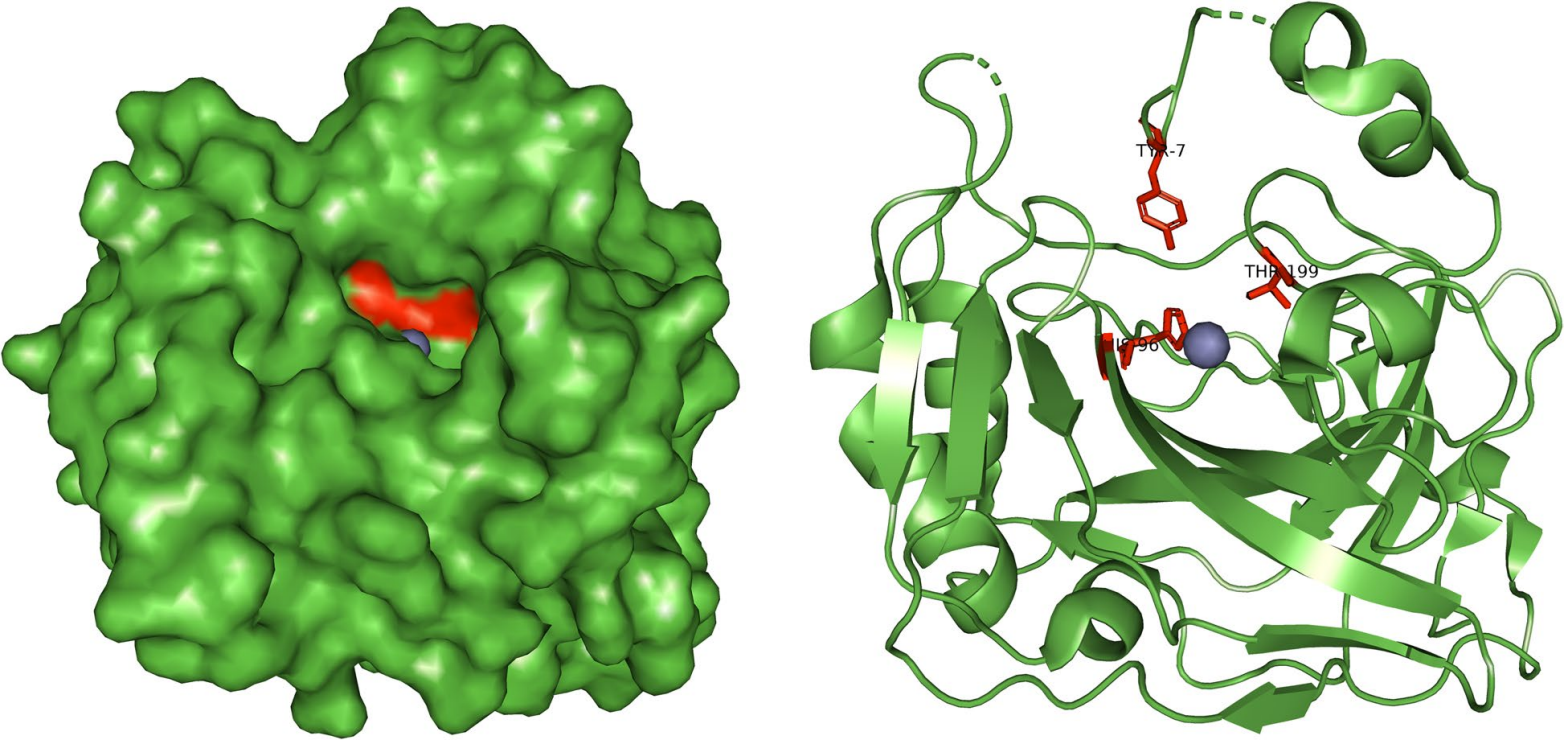
Amino acids that form the active site of human carbonic anhydrase

According to the research of a group of authors, the amino acids His96, Tyr7, and Thr199 are in the active site of carbonic anhydrase [35–44] and with this gridbox we tried to capture it. The mentioned amino acids can be seen in Fig. 7.

**Fig. 8:**
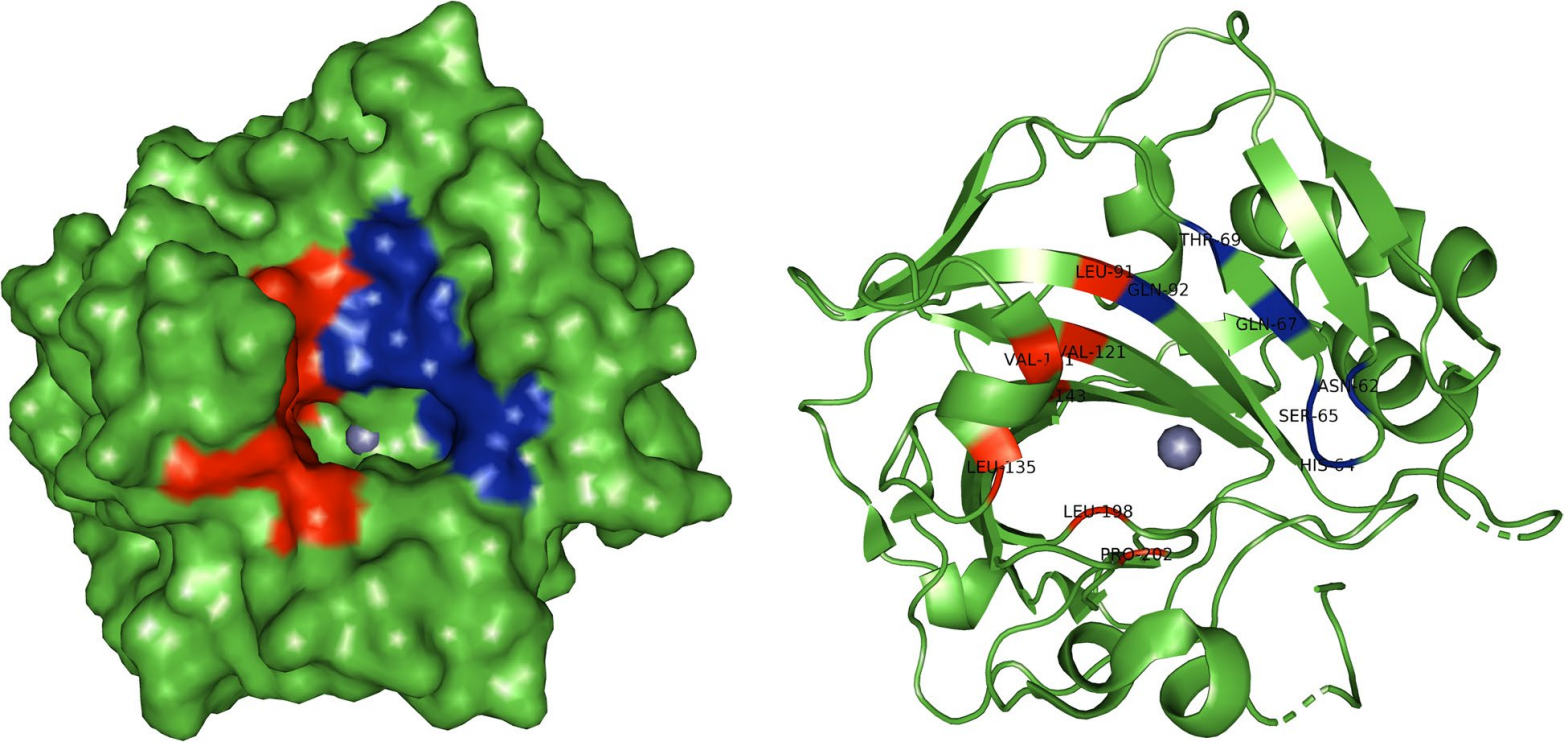
Visualization of the active site location

The active site of CA IX is located in a large conical cavity which is extending from the surface to the center of the protein. A zinc ion is located at the bottom of this cavity. Two different regions composed of hydrophobic or hydrophilic amino acids limit the active site. In particular, Leu-91, Val-121, Val-131, Leu-135, Leu-141, Val-143, Leu-198, and Pro-202 define the hydrophobic region, while Asn-62, His-64, Ser-65, Gln-67, Thr-69, and Gln-92 define the hydrophilic [43]. Figure 8 shows the region where the active site is located. The hydrophobic region is marked in red, while the hydrophilic region is marked in blue.

The results obtained with the docking analysis for halogenated boroxine and human carbonic anhydrase can be seen in Table 2. As mentioned before, the first result (−6.8 rmsd l.b. 0.000; rmsd u.b. 0.000) is the most relevant, while the other results are presented in relation to it. Also, the result under the ordinal number 2 (−6.6 rmsd l.b. 1.267; rmsd u.b. 1.405), which has RMSD values lower than 2 Å, is considered accurate and relevant. The values of other results are higher than 2 Å and they are considered irrelevant [33, 34].

**Table 2:** Results of the docking analysis in Autodock Vina for halogenated boroxine and human carbonic anhydrase

Mode	Affinity (kcal/mol)	Distance from best mode	
		Rmsd l.b.	Rmsd u.b.
1.	−6.8	0.000	0.000
2.	−6.6	1.267	1.405
3.	−6.2	4.014	5.391
4.	−6.1	3.728	5.164
5.	−6.0	11.923	12.188
6.	−5.9	5.931	6.820
7.	−5.9	2.629	2.948
8.	−5.9	11.523	11.937
9.	−5.8	12.626	13.001

**Fig. 9:**
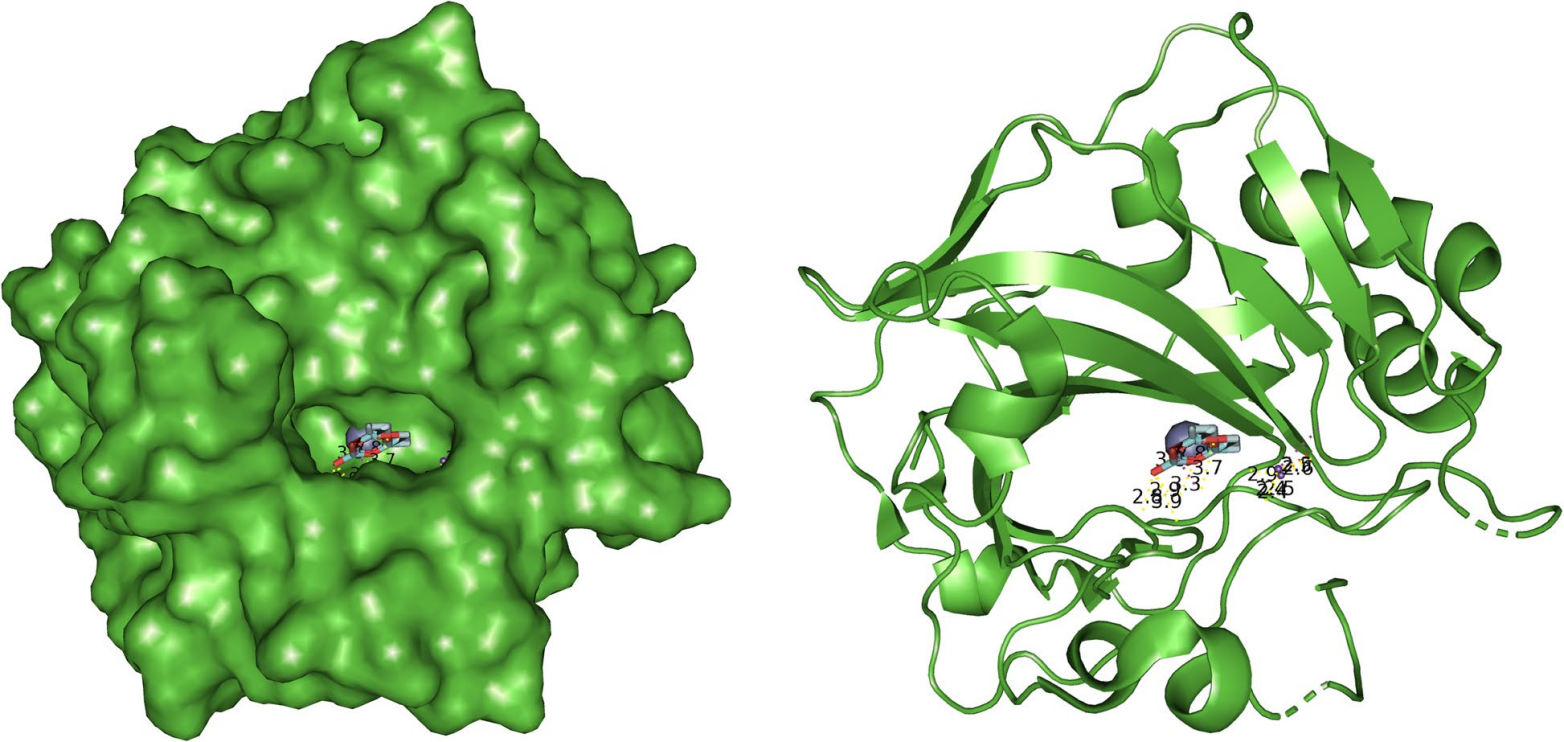
General overview of the interaction between halogenated boroxine molecule and human carbonic anhydrase IX

The results obtained from AutoDock Vina for the prediction of the interaction between halogenated boroxine and human carbonic anhydrase were visually analyzed in PyMOL. The visual display gives us a better insight into the interaction and position of the molecule in regard to the protein. The first result of the gridbox is presented in Fig. 9.

**Fig. 10:**
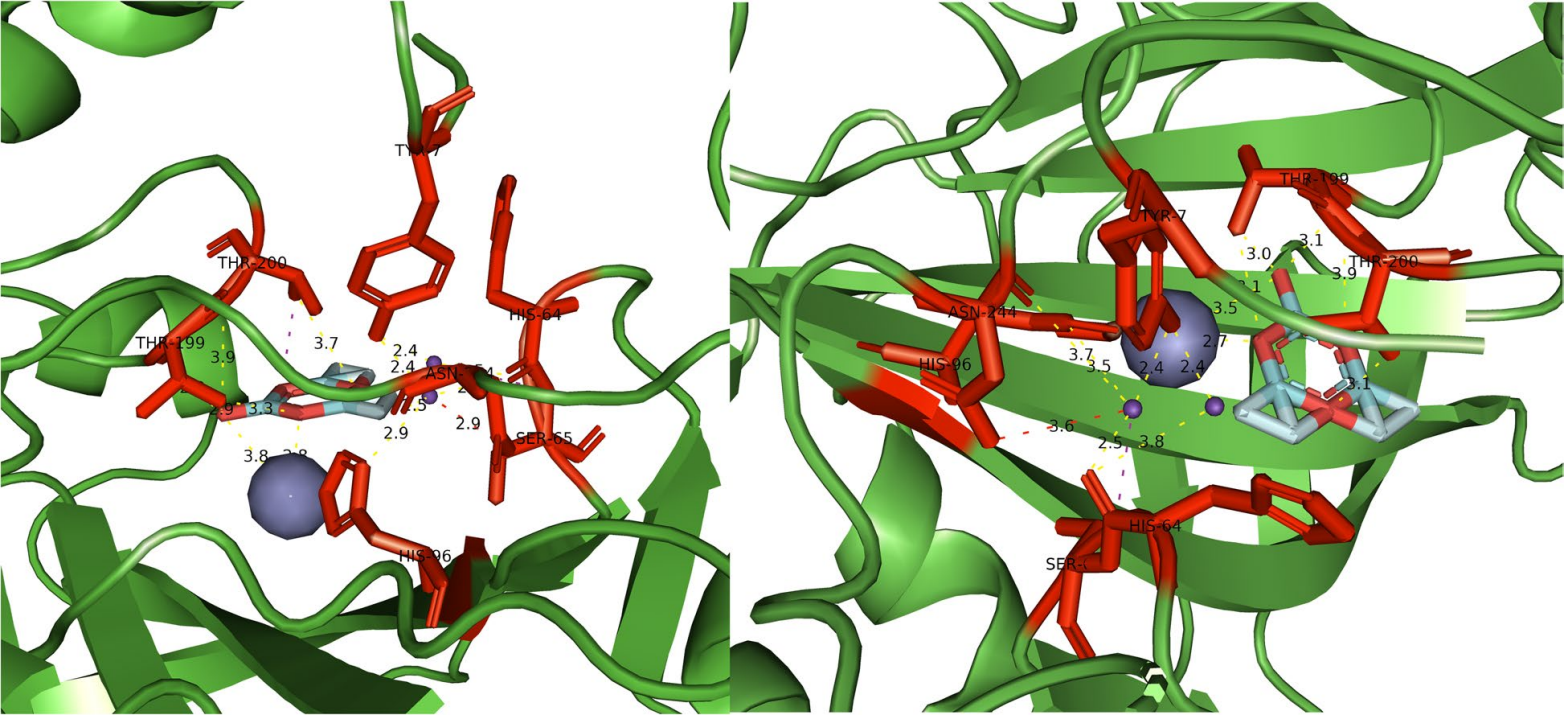
Closer look at the interaction between HB and hCA IX for the first (left) and the second (right) result

Figure 9 shows the interaction of halogenated boroxine and human carbonic anhydrase. The HB molecule is marked in a combination of red, green, blue, and purple, and the zinc ion is marked in grey, while the carbonic anhydrase structure is marked in green. This figure gives us an overall insight into the binding site of the molecule to the protein. Also, this shows that the molecule is near the active center of carbonic anhydrase, which was covered by the coordinates of the gridbox. Figure 10 will give us a closer look at the place of interaction for the two relevant results.

Figure 10 shows a closer view of the interaction between the HB molecule and the hCAIX protein for the first two results. The HB molecule is marked in combination of red, green, blue, and purple; the connections between atoms, i.e., the interaction between the molecule and the protein, are marked in yellow. The amino acids His96, Tyr7, and Thr199 are marked in red and it is visible that the binding sites are the amino acids of the active site. This confirms our assumption that halogenated boroxine reacts with human carbonic anhydrase in its active site and in that way blocks the binding of other elements to carbonic anhydrase.

By a detailed analysis of the structure of carbonic anhydrase, it can be concluded that the activity of p-nitrophenyl esterase and CO2 hydrase directly depends on the hidrophobicity of previously mentioned amino acids, and this amino acid property is considered the most important part for the catalytic role of enzymes [40]. Consequently, we can say that the analyzed and obtained results in our work also report that in the interaction of hCA IX and HB, CO2 binding also plays an important role.

## Conclusion

After analyzing the structural interaction between the molecule of halogenated boroxine and human catalase protein, we concluded that the molecule interacts with the CAT protein in its active site and reacts directly with heme prosthetic group, which confirmed our hypothesis. The results we obtained using the previously mentioned dimensions of the gridbox for CAT showed that the HB molecule reacts with the human catalase protein in its active center, i.e., in a pocket made of amino acids Tyr358, Arg354, His218, and Asp348, and the heme prosthetic group.

By analyzing the bond between structures, functions, and interactions, we aimed to provide evidence that the molecule of HB interacts with hCA IX in its active site. For carbonic anhydrase, these results served as evidence that there was an interaction with HB, where it was shown that HB reacts with hCA IX in its active site with His64, Tyr7, and Thr199 and in the area of the hydrophilic and hydrophobic regions consisting of previously mentioned amino acids.

We believe that these findings will significantly contribute to further research of the structural interactions of the halogenated boroxine molecule and will facilitate the understanding of its mechanism of action, which has not yet been accurately determined. The three-dimensional modeling of the HB molecule opens the way for many structural and bioinformatics research, which is the starting point for all major in vitro and in vivo studies of the reactions and mechanisms of action of this molecule.

## Data Availability

The datasets used and/or analyzed during the current study are available from the corresponding author on reasonable request.

## Abbreviations

Å: Ångstrom
CAT: Human catalase
CA: Carbonic anhydrase
HB: Halogenated boroxine
hCA: Human carbonic anhydrase
PDB ID: Protein data bank identifier
RCSB PDB: Research Collaboratory for Structural Bioinformatics Protein Data Bank
RMSD: Root-mean-standard-deviation
ROS: Reactive oxygen species
